# General Attitudes towards Artificial Intelligence Scale (GAAIS): Hungarian adaptation and links to personality traits

**DOI:** 10.3389/fpsyg.2025.1703750

**Published:** 2025-11-14

**Authors:** Sándor Rózsa, Szabolcs Bandi, István Hartung, Imre A. Török, Julia É. Varga, Eszter H. Somlai, Robert Herold, János Kállai

**Affiliations:** 1 Department of Personality and Health Psychology, Institute of Psychology, Károli Gáspár University of the Reformed Church in Hungary, Budapest, Hungary; 2 Department of Psychiatry and Psychotherapy, Medical School, University of Pécs, Pécs, Hungary; 3 Faculty of Health Sciences, University of Pécs, Pécs, Hungary; 4 Kiskunhalas Semmelweis Hospital, University Teaching Hospital, Psychiatry Unit, Kiskunhalas, Hungary; 5 Institute of Behavioral Sciences, Medical School, University of Pécs, Pécs, Hungary

**Keywords:** artificial intelligence, General Attitudes towards Artificial Intelligence Scale, reliability, validity, personality trait patterns

## Abstract

**Background:**

The present study undertook the adaptation and psychometric validation of the Hungarian version of the General Attitudes toward Artificial Intelligence Scale (GAAIS) to assess both positive and negative attitudes toward artificial intelligence (AI) in relation to psychosocial functioning and personality traits.

**Methods:**

The adaptation followed international test-adaptation standards, involving translation, back-translation, and expert review. A total of 704 participants (557 women, 144 men) aged 18–60 years (M = 27.8, SD = 10.6) completed the GAAIS together with several validated self-report measures: the Mental Health Continuum–Short Form (MHC-SF), Self-Concept Clarity Scale (SCCS), frequency of AI usage, Problematic Internet Use Questionnaire (PIUQ), and Schizotypal Personality Questionnaire–Brief Revisited (SPQ-BR).

**Results:**

The Hungarian version showed solid internal consistency (Cronbach’s *α* = 0.85 for the positive and 0.81 for the negative subscale) and a clear two-factor structure, supported by confirmatory factor analysis (CFI = 0.951, RMSEA = 0.058). The frequency of AI use in daily life emerged as the strongest predictor of both positive and negative attitude scores lending further support to the construct validity of the scale. The association analysis revealed that the behavioral components of AI-related attitudes are shaped by the competing motivational forces—approach (positive) and avoidance (negative). Specifically, the frequent use of AI is linked to the positive attitudes of GAAIS. In contrast, the unfavorable use of AI is associated with the negative attitudes of GAAIS. In the affective domain, anxiety sensitivity is associated with a negative attitude, and in the cognitive domain, schizotypal cognitive characteristics and difficulties in self-integration are linked to elevated negative attitudes in GAAIS. However, on the other pole of this cognitive dimension, adequate self-integration does not play a significant role in the formation of an AI-related positive attitude.

**Conclusion:**

These findings confirm the reliability and validity of the Hungarian GAAIS and highlight the importance of personality traits in shaping adaptive and maladaptive attitudes toward AI. The results underscore the value of a multidimensional framework for understanding AI attitudes. Adaptive traits were associated with psychological resilience, effective self-regulation, and constructive digital engagement, whereas maladaptive traits were correlated with social anxiety and problematic interactions with the internet and artificial intelligence (AI) technologies. A critical question remains: What outcomes may arise from when individuals hold positive attitudes toward AI but simultaneously experience difficulties with self-integration? This paradox highlights the need for further research into the complex interplay between personality structure and digital adaptation.

## 1 Introduction

### 1.1 Attitude towards artificial intelligence

Civil and professional discourse on social media frequently shapes public perceptions of artificial intelligence (AI), highlighting both positive and negative attitudes. Considering the widespread impact of emerging technologies and innovative learning approaches on our perception of both social and physical environments, this study focuses on the role of artificial intelligence within this evolving technological landscape. AI cannot be considered a conventional device, such as a desktop computer or smartphone. Unlike these tangible tools, AI systems operate through software that is spatially and temporally dispersed and often functions invisibly across multiple platforms. The abovementioned abstract and elusive qualities can evoke a wide spectrum of attitudes, ranging from enthusiasm to skepticism regarding the usability of AI. Ambivalent responses often amplify the uncertainty surrounding artificial intelligence (AI), raising concerns about its conscious controllability, contextual appropriateness, and potential behavioral consequences. These reactions may also trigger anxiety and foster negative beliefs about AI-driven outcomes. Following Allport (1935), “attitude” is defined simply as a person’s inherent tendency to react in a certain way, encompassing behaviors, emotions, and thoughts. Its function is to reduce variability in reactions, facilitate general approach or avoidance tendencies, and preserve self-functioning’s conceptual and temporal stability (Zhang and Dafoe, 2019). Accurately measuring attitudes toward AI presents several challenges. The following key questions arise: Are humans dominant or subordinate in their interactions with AI? Do AI-generated decisions have contextual validity? Where do the algorithms that underpin AI systems originate and how are they edited and deployed? Addressing these questions requires a combination of popular media insights, scientific analysis, and a systematic evaluation of the valence of public attitudes to establish reliable methods for assessing the motivational sources behind AI-related beliefs. The present study sought to investigate the statistical properties of the GAAIS, as developed by Schepman and Rodway (2020, 2023). This study examined the validity and reliability of the GAAIS within a Hungarian sample.

### 1.2 Measuring methods of AI

Several methods have been developed to measure AI attitudes. To understand attitudes toward AI, we draw upon the Unified Theory of Acceptance and Use of Technology (UTAUT), which examines how personality traits influence digital technology acceptance. This theoretical framework guides our methodology and analysis, ensuring a focused investigation into the Hungarian cultural and psychometric adaptation of the GAAIS. The degree of novelty seeking, openness, and negative emotions, such as worry, determine attitudes toward digital environments and AI (Barnett et al., 2015; Kortum and Oswald, 2018; Yoo, 2019; Ikkatai et al., 2022).

Multiple qualitative and quantitative methods are available to measure AI attitudes. Buck et al. (2022) conducted semi-structured interviews with general practitioners and revealed that attitude toward AI were predominantly favorable. However, the participants expressed a range of apprehensions, including existential anxiety, diagnostic inaccuracies, ethical misuse of data, changes in the dynamics of the doctor–patient relationship, external stakeholder pressures, and individualized contextual factors. Moreover, AI healthcare users lack knowledge of the safety and credibility of the source that provides AI-generated information.

In addition, Sindermann et al. (2021) developed an Attitude Toward Artificial Intelligence Scale (ATAI) in a Chinese study and revealed that AI acceptance was linked to traits of openness and agreeableness. However, the German study by Sindermann et al. (2022) did not identify similar associations. Individual behavioral tendencies related to AI use may also reflect underlying personality and self-functioning patterns. Conversely, AI often uses personal information from hidden sources and is difficult to verify; thus, a customized database and the machine’s conclusions may lead to negative consequences. Individuals are increasingly forced into decision-making when information floods. Schepman and Rodway (2020, 2023) developed a confirmed quantitative method and introduced a scale for measuring positive and negative attitudes toward artificial intelligence (GAAIS). They found that introverted individuals exhibited a positive attitude toward AI and underestimated its negative aspects. However, general trust endorses AI-related positive attitudes, whereas distrust manifests as a negative emotional state. Other Italian and Chinese versions of the GAAIS support the original version’s validity and suggest its adequate use in diverse cultures (Seo and Ahn, 2022; Kaya et al., 2024; Huang et al., 2025; Cicero et al., 2025).

Applying another scale (introduced and adapted to school children, Student Attitudes Toward AI, SATAI; Suh and Ahn, 2022; Hussain, 2020) found that positive attitudes toward digital technologies and machine equipment may include increased interest in novelty and positive expectations for usefulness, but at the same time, negative attitudes may frequently be detected due to lack of personal contact with AI. In addition, they showed that personal dispositions significantly influence accepting or rejecting attitudes, and the lack of a social environment that encourages the use of AI does not support the adaptation of AI in school training and common usage. These results (Barnett et al., 2015; Rheu et al., 2021) emphasize the specific role of introversion-related reduced social interaction, loneliness, constricted affects, and dominance of avoidance behavior in the navigation of the digital environment to use machinery technologies, specifically AI. Several AI-related attitude scales have been continuously developed (see the brief questionnaire-based version of the AIAS-4, Grassini, 2023).

As AI use is progressively spreading in different populations, the control of nationality and demographic variables demands the exploration of new test confirmation studies and new personality associations to understand the nature of AI adoption in individuals’ lives and careers (Kaya et al., 2024; Tien, 2024). It should clarify some basic AI-related questions: What information is related to me? What types of decisions and actions express my desires and characteristics? What attitudes are alien to me? Adequate responses to these questions require reliable self-report methods. The GAAIS, which has been used in various samples and nationalities (Montag et al., 2023, 2024; Naiseh et al., 2025) as well as in different theoretical frameworks and personality dimensions (Montag and Elhai, 2025), may be a potential candidate among the applied scales. The growing international interest in AI across cultural, scientific, governmental, and industrial sectors—alongside advances in AI measurement techniques, underscores the need for precise and standardized evaluation methods. Such frameworks are vital for fostering innovative interventions that enhance usability and mitigate both perceived and actual risks.

So far, there has not been an official adaptation or validation of an AI attitude scale in Hungarian. Consequently, this study constitutes the inaugural effort to deliver a psychometrically robust Hungarian adaptation of an internationally acknowledged instrument (GAAIS). This adaptation addresses a significant deficiency in the literature and facilitates subsequent research on AI-related attitudes within Hungarian-speaking populations. There are other tools that are used in other countries, like ATAI, AIAS-4, and SATAI, but none have been adapted or tested for use in Hungary.

### 1.3 Psychological adaptation and maladaptation

In contemporary public discourse, AI is often described as a technological capability that occasionally exceeds human cognitive capacity. However, its rapid evolution has raised concerns about its impact on cognitive development, interpersonal relationships, behavioral patterns, and psychological functioning, particularly concerning the formation of coherent self-states, attachment to significant others, and personal identity (Gnambs and Appel, 2019; Gillath et al., 2021). The degree to which individuals adapt or maladapt to AI is influenced by a complex interplay of demographic, economic, and sociocultural factors, as well as personal developmental history. The key determinants of successful adaptation include personality traits, habitual approaches to confronting or avoiding challenges, and self-regulatory capacity. These elements shape how individuals engage with AI and navigate its integration into daily life. Biologically rooted affective factors, such as emotional valence (positive or negative), sensitivity to uncertainty, fear or anxiety tendencies, and the capacity for conscious control – can significantly influence mental processes during critical decision-making moments. These factors affect whether individuals accept or reject new technologies or relationships in ambiguous or emotionally salient situations. The coherence, adequacy, and clarity of self-functions play pivotal roles in mediating internal and external environmental requirements. This is a foundation for developing either positive or negative attitudes toward novel experiences, especially when information is scarce or incomplete (Guidano and Liotti, 1983; Cicero, 2017). Furthermore, a stable self-concept and consistent temporal stability are essential for shaping attitudes and guiding responses to AI, regardless of whether those responses lean toward acceptance or resistance.

### 1.4 Objectives and hypotheses

This study aimed to conduct the cultural and psychometric adaptation and validation of the General Attitudes toward Artificial Intelligence Scale (GAAIS) using a Hungarian sample.

The theoretical reasoning for the study was grounded in well-established frameworks of technology acceptance, including the Unified Theory of Acceptance and Use of Technology (UTAUT; Venkatesh et al., 2003) and the Technology Acceptance Model (TAM; Davis, 1989). These models emphasize the influence of individual differences, perceived usefulness, and emotional responses on technology adoption. Extending these ideas, prior research has shown that self-concept clarity, anxiety sensitivity, and personality traits play important roles in shaping digital adaptation and attitudes toward technology (Petre, 2021; Stănescu and Romașcanu, 2024; Hinds and Joinson, 2024). Individuals with a coherent and stable self-concept are generally more open to innovation and experience less uncertainty in interacting with AI, whereas those with self-concept confusion or maladaptive personality features are more likely to respond with anxiety or avoidance. These perspectives provide a theoretical foundation for the current hypotheses linking self-functioning, mental health, and attitudes toward AI.

Building on prior research in this field (Schepman and Rodway, 2023; Kaya et al., 2024), we hypothesized that both positive and negative attitudes would emerge in the Hungarian context and that the valence of these beliefs would be linked to distinct personality trait patterns rooted in adaptive or maladaptive predispositions. We posited that self-concept clarity, stable self-functioning, and good mental health are positively associated with favorable attitudes toward AI and its integration. Conversely, individuals exhibiting self-dysfunction and facing challenges in adapting to both physical and digital environments are expected to exhibit more negative attitudes toward AI.

To assess indicators of adaptive and maladaptive functioning across emotional, cognitive, and behavioral domains, we measured anxiety, self-concept clarity, schizotypal traits, general maladaptation risk, problematic Internet use, and frequency of AI-related practices. The behavioral components included AI use frequency, the PIUQ subscales for obsessive use, neglect, and control disorder, and the SPQ behavioral disorganization factor. The affective components comprised the MHC measure of positive mental health, the ASI anxiety scores, and the SPQ interpersonal (affective) factor. The SCCS self-concept clarity scale and the SPQ cognitive factor represented cognitive components.

## 2 Methods

### 2.1 Participants and the procedure

A total of 713 participants were enrolled in the study; however, nine individuals were ruled out because GAAIS tests were not administered. A total of 704 healthy participants, including 557 females (79.1%, M = 27.1 years, SD = 10.1) and 147 males (20.9%, M = 30.6 years, SD = 12.3), were recruited through an advertisement. The accepted age range is 18–60 years. All participants were informed of the study objectives and provided written informed consent according to the principles outlined in the Declaration of Helsinki. Participation in the study was voluntary and unpaid. The sample comprised graduate and postgraduate students from regional universities, all of whom were members of a university campus community. Participants regularly used computers, the Internet, and social media platforms, primarily Facebook. The ETT TUKEB granted ethical approval for the study (approval number: BM16388-1/2023). Data were collected using a predetermined questionnaire package. While participants were allowed unlimited time, the completion process generally took approximately an hour.

### 2.2 Instruments

Sociodemographic data and AI usage: The sociodemographic information included gender, age, and educational level. Furthermore, a question related to participants’ experiences with AI systems in everyday life was presented later in the survey, immediately before the GAAIS. Participants were asked to indicate how frequently they used AI in their daily lives on a Likert scale ranging from 1 (never) to 5 (daily).

#### 2.2.1 The General Attitudes towards Artificial Intelligence Scale (GAAIS)

This instrument, developed by Schepman and Rodway (2020, 2023), consists of 20 items divided into two subscales: one measuring positive attitudes (12 items) and the other negative attitudes (eight items) toward artificial intelligence. Example of a positive attitude: “I am impressed by what AI can do.” Example of a negative attitude: “I think the artificial intelligence is sinister.” The responses ranged from “strongly disagree” (1) to “strongly agree” (5). For both scales of the GAAIS, mean scores were calculated by dividing the total score by the number of items on the respective scale. According to the International Test Commission (2017) guidelines, the GAAIS underwent cultural and psychometric adaptation, following a forward–backward translation procedure. The initial Hungarian version was produced by two independent translators. Subsequently, a bilingual professional unfamiliar with the original scale back-translated the items into English. The final version of the scale was reviewed and finalized by the translation team.

#### 2.2.2 Mental Health Continuum–Short Form (MHC-SF)

The MHC-SF (Keyes, 2002; Reinhardt et al., 2020) comprises 14 items. Participants used a 5-point Likert scale ranging from 0 (never) to 5 (every day). Mental health, including social adaptation, positive emotionality, psychological well-being, and overall positive mental health, is measured. A higher aggregated MHC-SF score indicated good mental health. The scale exhibited good internal consistency (Cronbach’s *α* = 0.89).

#### 2.2.3 Problematic Internet Use Questionnaire (PIUQ)

The PIUQ (Demetrovics et al., 2008; Koronczai et al., 2011) contains 18 items on three scales. The Obsession Scale measures obsessional preoccupation with Internet activities. The Neglect Scale assesses disregard for non-Internet activities. The Control Disorder Scale refers to the difficulty in controlling one’s Internet use. Participants used a 5-point Likert scale ranging from 1 (never) to 5 (almost always). A higher total score on the scale indicated a greater likelihood of Internet use and social maladjustment. The PIUQ demonstrated good internal consistency (Cronbach’s *α* = 0.89 to 0.91).

#### 2.2.4 Anxiety Sensitivity Index (ASI)

The ASI (Reiss et al., 1986; Kerekes, 2012) consists of 16 items. The participants used a 5-point Likert scale ranging from 1 (not disturbing) to 5 (very disturbing). The sensitivity to anxiety across somatic, cognitive, and social domains was measured using the ASI. A higher aggregated score indicates elevated vulnerability to interpersonal avoidance behavior and anxiety symptoms in various areas of personal and public life. The aggregated ASI score had an excellent internal structure (Cronbach’s *α* = 0.91).

#### 2.2.5 Self-Concept Clarity Scale (SCCS)

The SCCS (Campbell et al., 1996; Hargitai et al., 2020) comprises 12 items rated on a 5-point Likert scale, ranging from 1 (strongly disagree) to 5 (strongly agree). The SCCS assesses the self-concept clarity degree. A higher SCCS score indicated good mental health, greater self-functional stability, and a clear self-concept definition. The SCCS exhibited excellent internal consistency (Cronbach’s *α* = 0.91).

#### 2.2.6 Schizotypal Personality Questionnaire–Brief Revisited (SPQ-BR)

The SPQ-BR (Cohen et al., 2010; Kállai et al., 2018) was used to assess vulnerability to schizotypy associated with self-disorders. The instrument comprises three primary factors: Behavioral disorganization (eccentric behavior and odd speech) II. Affective-interpersonal (encompassing no close friends, constricted effect, and social anxiety). Cognitive (including ideas of reference, suspiciousness, magical thinking, and unusual perceptual experience). Responses were recorded using a 5-point Likert scale ranging from 0 (strongly disagree) to 4 (strongly agree), with higher total scores indicating greater difficulties in self-construction and increased social maladaptation. A high schizotypy score does not indicate a diagnosis of a schizotypal personality disorder in a healthy population. Rather, it reflects a tendency toward schizotypal-like traits characterized by developmental vulnerabilities in cognitive, affective, and behavioral deficits related to self-weakness. The internal consistency of the main factors was excellent (Cronbach’s *α* = 0.90 to 0.94).

### 2.3 Statistical analysis

Statistical analyses began with descriptive statistics and reliability checks for the GAAIS items and scales, including the examination of gender and age differences. Skewness and kurtosis were used to assess item and scale distributions, and Cronbach’s alpha was used to estimate internal consistency. Gender differences were tested using independent sample *t*-tests, and age was explored using Pearson’s correlations. To assess structural validity, exploratory factor analysis (EFA) was conducted using maximum likelihood estimation with oblique rotation. The number of factors to be retained was determined through parallel analysis, and polychoric correlations were used to account for the items’ non-normality.

Subsequently, Confirmatory Factor Analysis (CFA) tested the two-factor model of the GAAIS using Diagonally Weighted Least Squares (DWLS) estimation, which is suitable for non-normally distributed variables. In line with the initial validation study conducted by Schepman and Rodway (2023), we opted to primarily utilize model fit indices derived from DWLS estimation. This decision was based not only on the need for comparability, but also on what we learned from methodological literature that DWLS works well with ordinal data and often gives more stable estimates than other methods, like Weighted Least Squares (WLSMV) or Maximum Likelihood (ML) (Li, 2016; DiStefano and Morgan, 2014). In our study, DWLS yielded superior fit indices, while alternative methods often imposed more significant penalties on items with weaker loadings or cross-loadings – an issue particularly relevant in psychological scales. We also know how important it is to be honest when you report. As a result, we used model fit indices from the WLSMV and ESEM (Exploratory structural equation modelling) estimation methods, which is in line with recent research (e.g., Kaya et al., 2024). This makes it easier to compare things and will help with future studies that look at different cultures.

Model fit was evaluated with the Comparative Fit Index (CFI), Tucker-Lewis Index (TLI), Root Mean Square Error of Approximation (RMSEA), and Standardized Root Mean Square Residual (SRMR), applying commonly accepted cutoffs (Hu and Bentler, 1999): CFI > 0.90 (adequate) or >0.95 (good); RMSEA < 0.05 (good) or <0.08 (adequate); SRMR < 0.05 (good) or <0.10 (adequate).

Measurement invariance across gender was examined using a multigroup confirmatory factor analysis (MGCFA), following the general recommendations outlined by Vandenberg and Lance (2000) and Byrne (2013). Three increasingly constrained models—configural, metric, and scalar invariance—were tested to determine whether the factorial structure of the GAAIS was comparable for men and women. Model comparisons were based on changes in the Comparative Fit Index (CFI) and the Root Mean Square Error of Approximation (RMSEA), with differences of ≤0.010 for CFI and ≤0.015 for RMSEA interpreted as evidence of invariance (Cheung and Rensvold, 2002; Chen, 2007).

## 3 Results

### 3.1 Descriptive statistics and reliability

The descriptive statistics and distribution properties (skewness and kurtosis) of the GAAIS items are presented in Table 1. Notably, Item 5 (I am impressed by what AI can do). 14 (AI has many beneficial applications in Artificial Intelligence) yielded mean scores above 4 on a 1–5 Likert scale, reflecting strong endorsements from the respondents. Skewness and kurtosis values within ±1 are typically considered excellent, whereas values up to ±2 are still regarded as acceptable and not indicative of serious deviations from normality (Kline, 2011; Byrne, 2010; Hair et al., 2022). The majority of the 20 GAAIS items were within the ±1 range, indicating excellent distributional properties. Only a few items, specifically items 5, 12, and 14, exceeded this threshold, with item 14 showing a kurtosis of >3. Items 9 and 18 displayed marginal deviations with kurtosis values slightly above 1.

**Table 1:** Descriptive statistics, reliability, and loadings of CFA factors.

Item	M	SD	Skew-ness	Kurtosis	CFA loadings	
					*Positive*	*Negative*
1. For routine transactions, I would rather interact with an artificial intelligence system than with a human.	2.29	1.15	0.49	−0.86	0.49	
2. Artificial Intelligence can provide new economic opportunities for this country.	3.22	0.99	−0.46	−0.21	0.59	
3. Organisations use artificial intelligence unethically.	3.22	0.90	−0.17	0.07		0.32
4. Artificially intelligent systems can help people feel happier.	2.56	1.08	0.23	−0.86	0.48	
5. I am impressed by what artificial intelligence can do.	4.01	1.01	−1.15	1.09	0.50	
6. I think artificially intelligent systems make many errors.	3.41	0.86	−0.27	−0.18		0.13
7. I am interested in using artificial intelligence systems in my daily life.	3.33	1.09	−0.66	−0.30	0.69	
8. I find artificial intelligence sinister.	3.40	1.16	−0.35	−0.76		0.84
9. Artificial Intelligence might take control of people.	2.90	1.21	−0.05	−1.01		0.58
10. I think artificial intelligence is dangerous.	3.38	1.15	−0.47	−0.63		0.83
11. Artificial Intelligence can have positive impacts on people’s well-being.	3.16	1.01	−0.47	−0.49	0.63	
12. Artificial Intelligence is exciting.	3.84	0.95	−1.12	1.45	0.68	
13. An artificially intelligent agent would be better than an employee in many routine jobs.	3.68	1.05	−0.89	0.23	0.44	
14. There are many beneficial applications of artificial intelligence.	4.14	0.74	−1.29	3.60	0.52	
15. I shiver with discomfort when I think about future uses of artificial intelligence.	3.37	1.18	−0.39	−0.77		0.79
16. Artificially intelligent systems can perform better than humans.	2.89	1.05	0.07	−0.63	0.24	
17. Much of society will benefit from a future full of Artificial Intelligence	2.99	1.00	−0.07	−0.60	0.67	
18. I would like to use artificial intelligence in my own job.	2.98	1.24	−0.09	−1.04	0.77	
19. People like me will suffer if artificial intelligence is used more and more.	2.57	1.08	0.32	−0.46		0.66
20. Artificial intelligence is used to spy on people	2.70	1.09	0.09	−0.66		0.43
*Positive general attitudes towards AI*	3.26	0.63	−0.51	0.56		
*Negative general attitudes towards AI*	3.12	0.71	−0.22	−0.26		

The scores on the positive and negative subscales of the GAAIS were within acceptable ranges. When comparing scores by gender, males scored significantly higher [*t* (701) = 2.29, *p* = 0.022] and lower [*t* (701) = 2.10, *p* = 0.036] on the positive attitude scale than females. The correlation between the two subscales was moderate and negative (*r* = 0.35, *p* < 0.001). Age had a weak but significant positive association with positive attitudes (*r* = 0.084, *p* = 0.027), whereas age and negative attitudes had no significant relationship.

The positive attitude scale had good internal consistency (Cronbach’s *α* = 0.85) and all item-total correlations were above the recommended threshold of 0.20. The lowest correlation was observed for item 16 (*r* = 0.29). In contrast, the negative attitude scale yielded a Cronbach’s *α* of 0.81, with most item-total correlations being adequate; however, item 6 showed a relatively weak correlation of 0.10.

### 3.2 Exploratory and confirmatory factor analysis of the GAAIS

To determine the optimal number of factors to extract, a parallel analysis was conducted prior to the exploratory factor analysis (EFA). The confirmatory factor analysis (CFA) was then performed as a confirmatory re-analysis on the same dataset to test the robustness of the factor structure identified in the EFA. Polychoric correlations were computed, given the ordinal nature of the items and the non-normality observed in their distributions. This analysis suggests a two-factor GAAIS structure. The Kaiser–Meyer–Olkin measure of sampling adequacy (0.872) and Bartlett’s test of sphericity (*χ*^2^ = 6399.00, df = 190, *p* < 0.001) indicated that the data were suitable for factor analysis. Subsequent exploratory factor analysis with oblique rotation produced a two-factor solution, with most items loaded on their hypothesized factors. However, Item 6 of the Negative Attitude Scale showed a notably low factor loading of 0.07, which falls well below the commonly accepted threshold of 0.40.

Given the distributional properties of the GAAIS items, we applied a Diagonally Weighted Least Squares (DWLS) estimator for confirmatory factor analysis (CFA). This estimator is particularly appropriate because it is robust to multivariate normality violations and is well suited to ordinal or non-normally distributed data (Li, 2016; Mindrila, 2010). Confirmatory factor analysis yielded acceptable fit indices (*χ*^2^ (169) = 566.056, *p* < 0.001, *χ*^2^/df = 3.35, CFI = 0.951, TLI = 0.944, RMSEA = 0.058, and SRMR = 0.070), although three items (Items 3, 6, and 16) showed standardized factor loadings were below the recommended threshold of 0.40 (Table 1).

Model fit indices obtained using alternative estimation methods were weaker compared to those derived from DWLS. Specifically, WLSMV estimation resulted in *χ*^2^ (169) = 1333.883, *p* < 0.001, *χ*^2^/df = 7.89, CFI = 0.892, TLI = 0.878, RMSEA = 0.099, SRMR = 0.069; and ESEM estimation yielded *χ*^2^ (151) = 966.360, p < 0.001, *χ*^2^/df = 6.39, CFI = 0.924, TLI = 0.905, RMSEA = 0.088, SRMR = 0.057. These differences are likely due to the fact that WLSMV tend to penalize items with lower factor loadings more strongly, which can reduce overall model fit unless such items are excluded.

In our case, Items 3, 6, and 16 showed loadings below the recommended threshold (<0.40; see Table 1). Nevertheless, considering the sample size and the theoretical relevance of these items, we decided to retain them to maintain consistency with the original GAAIS structure and to preserve content validity. This approach is also in line with the original validation study (Schepman and Rodway, 2023), where similar item-level issues (e.g., Item 6) were observed and no exclusions were made. The implications of potential item removal will be further examined in a forthcoming cross-cultural investigation including data from multiple countries.

Finally, the multigroup CFA confirmed that the two-factor structure of the GAAIS was stable across gender. The differences in model fit between the configural and metric models (ΔCFI = −0.001, ΔRMSEA = 0.001) and between the metric and scalar models (ΔCFI = −0.001, ΔRMSEA = 0.001) were minimal, indicating that the factor loadings and intercepts can be considered equivalent for men and women. Overall, these findings support full measurement invariance and suggest that the GAAIS functions comparably across gender groups.

Correlation analysis (Table 2) revealed a strong inverse relationship between positive and negative attitudes, as measured by the GAAIS. Specifically, individuals exhibiting a highly positive attitude toward AI tend to show a markedly low negative attitude and vice versa. Further analysis indicated that a positive attitude toward AI was positively associated with good mental health and frequent usage of AI. However, the negative AI-related attitude is associated with lower self-concept clarity, lower AI usage frequency scores, and higher anxiety, schizotypal cognitive disorder, behavioral disorganization, and high scores in obsessions related to problematic internet use.

**Table 2:** Descriptive statistics and correlation matrix between positive and negative AI-related attitudes and other, adaptive, and maladaptive trait variables (controlled by sex and age).

	GAAIS		
	Mean (SD)	Positive attitude	Negative attitude
General Attitudes towards Artificial Intelligence Scale (GAAIS)			
Positive attitude	3.26 (0.63)		
Negative attitude	3.12 (0.71)	−0.38**	
Mental Health Continuum—Short Form (MHC-SF)	44.8 (12.0)	0.19**	−0.07
Self-Concept Clarity Scale (SCCS)	44.1 (10.3)	−0.02	−0.19**
Frequency of AI usage^a^	3.1 (1.3)	0.48**	−0.14*
Anxiety Sensitivity Index (ASI)	19.1 (11.4)	−0.05	0.19**
Schizotypal Personality Questionnaire (SPQ-BR)			
Cognitive	16.4 (9.2)	0.04	0.30**
Interpersonal affective	14.2 (8.2)	−0.06	0.11
Behavioral disorganized	12.3 (6.5)	−0.04	0.24**
Problematic Internet Use (PIUQ)			
Obsession	12.0 (5.3)	0.01	0.29**
Neglect	11.4 (4.0)	0.03	0.02
Control disorder	13.3 (4.4)	0.02	0.12

*p* < 0.01; **p < 0.05*.

a , *n* = 260.

## 4 Discussion

The presented psychometric results supported the original two-factor structure of the GAAIS, which was proposed by Schepman and Rodway (2020, 2023), which has also been confirmed by adaptation studies conducted in several other countries, such as Germany (Sindermann et al., 2022), China (Huang et al., 2025), Italy (Cicero et al., 2025), Korea (Seo and Ahn, 2022), and Turkey (Kaya et al., 2024). The present findings further reinforce previous observations that certain items (e.g., Item 6) show a weaker alignment with the intended scale structure. This is not unexpected, as positively and negatively worded items often qualitatively capture different aspects of attitudes toward AI, which may contribute to factor loading inconsistencies. The measurement invariance analysis further confirmed that the GAAIS performs equivalently across gender, supporting the interpretation that the observed gender differences in AI attitudes reflect true mean-level variations rather than measurement bias.

Studying the demographic variables, similar to other investigations using various methods (Liang and Lee, 2017; McClure, 2017; Naiseh et al., 2025), males scored significantly higher on positive attitudes toward AI than females. These gender differences can be regarded as genuine mean-level variations rather than the result of measurement bias, as the multigroup CFA provided clear evidence of full measurement invariance across gender. The correlation between the positive and negative scales was negative, similar to the results previously reported by Schepman and Rodway (2020, 2023). Age showed a significant positive association with positive attitudes toward AI; however, a considerable association between age and negative attitudes was not found in this sample. The expected missing difference between age and negative AI attitude is due to the specificity of the present sample, particularly the elderly and postgraduates with adequate digital competency.

AI attitudes can be interpreted using a different framework. In this study, the role of self-regulation in the intensity of positive and negative AI-related attitude formation was investigated. Behavioral, affective, and cognitive personality predispositions were theoretically selected from the available options. These traits reflect potential adaptive and maladaptive responses to a general digitally constructed synthetic environment. The three trait patterns of the cognitive, affective, and behavioral attitude components were analyzed. The frequency of AI, problematic Internet use, and the self-regulation-related general behavioral disorganization factor from the schizotypal traits represented the behavioral components. The affective component involves positive mental health, anxiety, and interpersonal and affective components related to schizotypal trait predispositions related to positive or negative self-regulation. The cognitive components included self-concept clarity and schizotypy cognitive factors. This study assumed that positive and negative attitudes play different roles in adapting to digital environments. The results indicate that negative attitudes toward AI are connected to maladaptive behavioral, cognitive, and emotional factors; however, positive attitudes are linked to adaptive and pragmatic affective and behavioral traits.

Our findings on the behavioral component of AI-related attitudes suggest that a positive attitude toward AI is associated with frequent use of AI and higher competency. Conversely, negative attitudes were related to adverse AI experiences and lower AI use activity. These results align with prior research conducted using a similar, albeit simpler, methodology in Arabic and UK samples, which demonstrated a positive correlation between AI-related competencies and well-being and a negative attitude toward low competency levels (Naiseh et al., 2025; Cicero et al., 2025). Importantly, our data suggest that positive past experiences with AI may foster more favorable attitudes toward its future and general use. This positive association is further supported by the affective dimension of well-being: individuals with a positive attitude toward AI tend to report better mental health. However, a link between negative attitudes and poor mental health was not observed.

The second group of findings focused on the affective dimension of attitudes toward the use of AI. Correlation analyses indicated that the affective components of schizotypal trait predispositions may serve as biopsychological contributors to the development of negative AI attitudes. These attitudes are shaped by negative affectivity and are linked to social anxiety, loneliness, constricted emotional expressions, and a general sense of interpersonal maladaptation. Using the ATAI framework, similar findings by Montag et al. (2024) suggest that negative affectivity rooted in biologically based separation anxiety reflects heightened sensitivity to novel and uncertain environments. Such environments, including AI, may act as catalysts for negative emotional responses, social withdrawal, and anxiety.

Our results support the interpretation that a biologically driven fear of attachment loss coupled with an intrinsic need for environmental security is one facet of negative attitudes toward AI. Individuals experiencing this response tend to perceive AI as an intangible and disembodied entity, making it difficult to establish emotional attachment without real positive interactions. Drawing on the principles of human and machine learning (Skinner, 1950; Du et al., 2025), we propose that a sustained sequence of positive experiences may facilitate the psychological embodiment of AI. In turn, this process could alleviate feelings of loneliness and reduce separation anxiety, positioning AI as a potential agent in mitigating such emotional distress. The implications of these results point to a dual trajectory: on the one hand, fostering a rational and constructive engagement with AI; on the other hand, risking the emergence of excessive dependency, driven by the pursuit of increasingly rewarding interactions, is a dual trajectory. Balancing these outcomes is essential for promoting healthy human-AI relationships.

A similar association between negative affectivity and negative attitudes toward the use of digital tools and modified environments – assessed through the Five-Factor Model of personality has also been reported (Barnett et al., 2015). Thus, AI technologies may function as virtual partners or transitional objects, thereby enabling the formation of surrogate relationships. These personalized, digitally controlled environments may expose vulnerabilities in self-functioning and underscore the weakness of the self and the limitations of emotional regulation, particularly among individuals predisposed to negative affectivity in technologically mediated settings. Schepman and Rodway (2023) highlighted that introversion is linked to elevated positive and negative attitudes toward AI. In contrast, our current study employs only indirect indicators of introversion – specifically, schizotypy-related negative affectivity – and reveals that this trait is associated with negative attitudes toward AI but not with positive ones. Previous findings on cognitive and affective biases in introverted individuals suggest a pronounced sensitivity to interpersonal events. Introverts typically exhibit higher baseline arousal than extroverts. This heightened arousal may escalate to anxiety and cognitive dysfunction when faced with socially ambiguous situations. Introverts often adopt avoidance strategies to mitigate such vulnerability, steering clear arousal-inducing scenarios to evade anticipated interpersonal punishment. In contrast, extraverts possess lower baseline arousal and actively seek stimulating environments, particularly those that offer interpersonal rewards (Eysenck, 1994; Mitchell and Kumari, 2016). Elevated arousal in introversion-like individuals may activate a socially reinforced anticipation of rewards in uncertain contexts. This mechanism, driven by the broadly appealing and socially endorsed nature of AI, could influence problem-solving strategies, enabling ambivalent yet controlled engagement with uncertain situations despite the underlying negative affectivity.

The third group of findings examined the role of cognitive components in shaping attitudes toward AI. Cognitive variables such as self-concept clarity and schizotypy-related cognitive traits did not significantly contribute to the formation of positive attitudes toward AI in this sample. However, association analyses revealed that negative attitudes were more pronounced among individuals exhibiting conceptual and temporal self-instability, cognitive lability, and cognitive disturbances. These include elevated magical thinking, heightened suspiciousness, increased interest in unusual perceptual experiences, intensified fantasy activity, elevated immersive tendencies, and impaired reality monitoring. Through visual, auditory, verbal, and scenic simulations, AI technologies emulate human cognition, offering seductive representations of reality that can draw individuals into an immersive engagement. In this context, immersion refers to a cognitive state in which critical judgment is suspended and the individual becomes absorbed in unfolding events while passively experiencing them without exerting agency or control (Tamás et al., 2022; Massaro et al., 2023). This immersive state plays a crucial role in embodied cognition, enabling individuals to access an experience’s emotional, conceptual, moral, and artistic dimensions (Riva, 2025). When an event’s temporal and spatial dynamics activate the self-representation system, the information becomes personalized and deeply integrated. While immersion can enhance cognitive and emotional engagement, it may also induce risks in individuals with impaired or unstable self-functioning. Immersion may lead to self-loss, craving, and psychological vulnerability in such cases (Truzoli et al., 2016). These risks serve as warning signals to individuals with pronounced cognitive schizotypal traits. Despite their capacity for immersive engagement, they may adopt a negative attitude toward AI as a protective mechanism to avoid the perceived threats of personal dissolution and psychological imminence. Fear of self-loss may represent a stable underlying motivation for this subgroup to resist AI use.

Our examination of gender and age correlation data in relation to the GAAIS scales revealed that females exhibited stronger negative attitudes toward AI than males, while males demonstrated more positive attitudes than females. These results partly support previously reported data (Schepman & Rodway, 2023). However, in the current sample, associations were only found between females and elevated negative attitudes toward AI; the corresponding positive attitude among males was not statistically supported. Regarding age, a significant positive association was found between older age and favorable attitudes toward AI. This finding contrasts with earlier research suggesting that younger individuals tend to hold more positive views toward digital technologies than older adults (Zhang and Dafoe, 2019). Nonetheless, the rapid proliferation and integration of digital technologies may have diminished the moderating effects of gender and age over time (Hauk et al., 2018; Mariano et al., 2022). As noted by Mariano et al. (2022), these demographic factors now play a more limited role in shaping attitudes toward AI. This unexpected pattern may stem from the unique characteristics of our sample, that is, the senior postgraduate participants, who are affiliated with a university campus, maintain an active engagement with digital learning and working environments and possess a high level of competence in using various digital tools in their daily lives.

In summary, data from the GAAIS, which measures attitudes toward AI, revealed a dichotomy. Positive attitudes are predominantly linked to adaptive personality traits, whereas maladaptive patterns tend to lead to negative attitudes. These maladaptive traits associated with negative AI beliefs are rooted in learned behavioral habits and are shaped by biologically and psychologically driven affective (interpersonal) and cognitive deficits. The impact of these traits, whether constructive or harmful, largely depends on an individual’s current stage of personality development. This includes the maturity of self-regulation capacities such as impulse control, emotional modulation, and self-functioning conceptual and temporal coherence. Our findings suggest that individuals with negative attitudes toward AI often engage in excessive rumination (obsessional ideas) about Internet use and struggle to suppress their urge to go online. Moreover, the activity control rate of usage is difficult when they are online, which would otherwise help mitigate the harmful effects of excessive internet use. Motivational drivers behind this behavior include vivid imagination, heightened suspicion, openness to atypical experiences, and magical expectations of positive outcomes. This constellation of schizotypal traits reflects a motivational conflict between approach and avoidance behaviors, in which potential negative consequences are frequently overlooked or dismissed. Such uncontrolled sensitivity to imagined ideas, distorted perceptions, and unrecognized risks fosters a vulnerable and defenseless self-state (Demetrovics et al., 2022; Kállai et al., 2021). The anticipated sense of virtual interpersonal safety and reinforcement of behavioral goals contribute to the persistence of these trait patterns within this ambivalent psychological landscape. Individuals with pronounced schizotypal tendencies may evolve into an “autistic home,” an interpersonally isolated virtual space that accommodates and perpetuates their unique cognitive-affective profile.

### 4.1 Limitations

This Hungarian sample consisted mainly of senior and junior postgraduate participants with advanced educational backgrounds, embedded within a university-specific digital culture and usage habits. The findings indicate that the GAAIS performs as a valid and reliable measure within this population. However, larger and more culturally diverse samples are needed to further substantiate the scale’s psychometric robustness and to confirm the generalizability of these results. Such extensions would enhance the broader applicability of the GAAIS and support its use in diverse international contexts.

Another limitation concerns the retention of several items with factor loadings below the conventional threshold (Items 3, 6, and 16). These items were retained to preserve theoretical coherence and consistency with the original GAAIS structure; nevertheless, future studies using larger and cross-cultural samples should re-examine their psychometric performance and consider potential item refinement.

Finally, the study was not pre-registered, which may somewhat limit the transparency and reproducibility of the research process.

## 5 Conclusion

The frequency of artificial intelligence use in everyday activities appears to be the most reliable predictor of scores on the GAAIS positive and negative attitude scales. These findings offer robust empirical support for the construct validity of the GAAIS. Underlying personality predispositions play a crucial role in shaping these attitudes, revealing the presence of latent, biopsychological anchored trait configurations—resembling psychotypy—that influence whether individuals develop favorable or unfavorable views toward AI. These traits modulate the impact of social anxiety, maladaptive behaviors, and diminished self-coherence and integration capacities, thereby amplifying or attenuating the emotional valence of AI-related attitudes. Such predispositions may also contribute to impairments in verbal communication and behavioral regulation, often manifesting in social interaction and decision-making disordered patterns. Conversely, a positive attitude toward AI—indicative of adaptive engagement with digital technologies—is associated with improved mental health, rational decision-making, and frequent, constructive use of AI. This contrast underscores a dichotomous or ambivalent pattern rooted in personality structure. Adaptive traits are correlated with more effective self-regulation, psychological resilience, and healthy digital behavior, whereas maladaptive characteristics are linked to social anxiety and problematic interactions with the Internet and artificial intelligence (AI) technologies. However, a critical question remains unresolved: what outcomes can be anticipated when an individual exhibits a positive attitude toward AI but simultaneously struggles with self-integration difficulties? Moreover, there are individuals for whom the evolution of their preferences remains unpredictable in the ongoing competition between the physically tangible world and the digitally constructed reality shaped by artificial intelligence.

## Data Availability

The datasets presented in this study can be found in online repositories. The names of the repository/repositories and accession number(s) can be found at: https://osf.io/wevcm.
